# Down-regulation of PPARgamma1 suppresses cell growth and induces apoptosis in MCF-7 breast cancer cells

**DOI:** 10.1186/1476-4598-7-90

**Published:** 2008-12-05

**Authors:** Yekaterina Y Zaytseva, Xin Wang, R Chase Southard, Natalie K Wallis, Michael W Kilgore

**Affiliations:** 1 Department of Molecular and Biomedical Pharmacology, University of Kentucky College of Medicine, 800 Rose Street, Room MS-305, Lexington, KY 40536-0298, USA

## Abstract

**Background:**

Peroxisome proliferator-activated receptor gamma (PPARγ) is a member of the nuclear hormone receptor superfamily and is highly expressed in many human tumors including breast cancer. PPARγ has been identified as a potential target for breast cancer therapy based on the fact that its activation by synthetic ligands affects the differentiation, proliferation, and apoptosis of cancer cells. However, the controversial nature of current studies and disappointing results from clinical trials raise questions about the contribution of PPARγ signaling in breast cancer development in the absence of stimulation by exogenous ligands. Recent reports from both *in vitro *and *in vivo *studies are inconsistent and suggest that endogenous activation of PPARγ plays a much more complex role in initiation and progression of cancer than previously thought.

**Results:**

We have previously demonstrated that an increase in expression of PPARγ1 in MCF-7 breast cancer cells is driven by a tumor-specific promoter. Myc-associated zinc finger protein (MAZ) was identified as a transcriptional mediator of PPARγ1 expression in these cells. In this study, using RNA interference (RNAi) to inhibit PPARγ1 expression directly or via down-regulation of MAZ, we report for the first time that a decrease in PPARγ1 expression results in reduced cellular proliferation in MCF-7 breast cancer cells. Furthermore, we demonstrate that these changes in proliferation are associated with a significant decrease in cell transition from G_1 _to the S phase. Using a dominant-negative mutant of PPARγ1, Δ462, we confirmed that PPARγ1 acts as a pro-survival factor and showed that this phenomenon is not limited to MCF-7 cells. Finally, we demonstrate that down-regulation of PPARγ1 expression leads to an induction of apoptosis in MCF-7 cells, confirmed by analyzing Bcl-2 expression and PARP-1 cleavage.

**Conclusion:**

Thus, these findings suggest that an increase in PPARγ1 signaling observed in breast cancer contributes to an imbalance between proliferation and apoptosis, and may be an important hallmark of breast tumorigenesis. The results presented here also warrant further investigation regarding the use of PPARγ ligands in patients who are predisposed or already diagnosed with breast cancer.

## Background

Breast cancer is the most common malignancy and the second leading cause of cancer related death among American women [1]. Despite of the fact that recent research efforts have significantly improved the outcome of breast cancer, the complexity and heterogeneity of this disease still urges the necessity to explore new and more specific drug targets. Peroxisome proliferator-activated receptor gamma (PPARγ), a member of the nuclear-hormone receptor family, has shown potential as a therapeutic target for prevention and treatment of breast cancer. PPARγ is a ligand-activated transcription factor. There are two isoforms of PPARγ protein, PPARγ1 and PPARγ2, the latter of which has the addition of 30 N'-terminal amino acids as a result of the usage of a different promoter and alternative splicing [2]. PPARγ plays an important role in adipocyte differentiation, insulin sensitivity, energy metabolism, immune response, and the development of the nervous system [3-5]. It is predominantly expressed in adipose tissues; although, it is also detected in various tissues such as cardiac and skeletal muscle, intestine, vascular smooth muscle, lung, breast, colon, and prostate [6,7]. Some polyunsaturated fatty acids [8-10] and arachidonic acid metabolites [11] are considered to be the natural ligands of PPARγ. Synthetic ligands of PPARγ include the thiazolidinedione class of anti-diabetic drugs (TZDs) such as rosiglitazone, pioglitazone, troglitazone [12,13], some non-steroidal anti-inflammatory drugs (NSAID) [14], and non-thiazolidinedione tyrosine [15]. In addition, a ligand-independent mechanism of PPARγ activation has also been observed due to altered phosphorylation status of the receptor [16].

Recently, PPARγ has emerged as a promising target for cancer therapy based on the fact that its activation by synthetic ligands such as TZDs have been shown to induce cell cycle arrest, apoptosis and differentiation in many human malignancies [17,18]. Several studies have demonstrated that PPARγ activation by agonists can promote growth inhibition and apoptosis in both primary and metastatic breast malignancies [19-22]. In addition to the anti-proliferative and pro-apoptotic effects, PPARγ ligands have also been reported to inhibit invasion and metastasis of human breast cancer cells [23,24]. However, these results were questioned by several studies that demonstrated the ability of PPARγ ligands to elicit anti-tumor effects via PPARγ-independent pathways and in the absence of PPARγ receptors [25,26]. Moreover, there is a debate that the concentrations of PPARγ ligands used in many studies are above the saturation level of the receptor. In fact, Roziglitazone, a widely studied PPARγ agonist, has shown to induce opposing effects when used in low versus high doses [27]. Furthermore, PPARγ antagonists have also shown anticancer effects in a wide range of epithelial cancer cell lines, usually with greater potency than agonists [28].

Existing data from *in vivo *studies is also controversial. Recent animal studies have demonstrated that PPARγ agonists can prevent mammary carcinogenesis and reduce the development of tumors in nude mice [29]. In contrast, another study has demonstrated an increase in the number of tumors when PPARγ ligand was administered [30]. To clarify the controversy arising from the use of pharmacological approaches, several animal studies utilized techniques that allowed evaluation of the consequences of PPARγ transactivation in breast cancer independent of exogenous stimulation. Studies which employed a genetic approach to explore the intrinsic role of PPARγ signaling have demonstrated that an increase in PPARγ signaling accelerates mammary gland tumor development and constitutive over-expression of PPARγ increases incidence of breast cancer in mice already susceptible to the disease [31]. This group has also shown that mice heterozygous for a null PPARγ mutation develop tumors with the same kinetics as those that carry two functional copies [31]. Furthermore, the ablation of PPARγ expression in the mouse mammary gland using a Cre- Lox recombination system has demonstrated that no tumors developed in mammary glands lacking PPARγ suggesting that PPARγ is not a tumor suppressor [32]. In summary, these observations suggest that reduced PPARγ expression does not contribute to the initiation of breast cancer; however, acceleration of PPARγ signaling after tumor initiation markedly promotes breast cancer development.

In this study, we have begun to elucidate the functional significance of endogenous PPARγ activation in breast cancer using an *in vitro *model. We have previously reported that PPARγ1, not PPARγ2, is expressed in normal mammary epithelial cells and breast cancer cell lines [33]. Our lab and others have also demonstrated that the level of PPARγ1 expression is significantly higher in breast cancer cell lines as compared to normal epithelial cells [33-36]. In addition, we have shown that a distinct promoter regulates PPARγ1 expression in MCF-7 cells and that *promoter switching *mediates differential PPARγ1 expression levels between normal and cancer cells [33]. The Myc-associated zinc finger protein (MAZ) has been identified as a transcriptional mediator of PPARγ1 in MCF-7 cells [37]. MAZ is a transcriptional factor that controls the expression of various genes through interactions between GC-rich DNA binding sites within the promoter sequence of target genes and the carboxyl-terminal zinc finger motifs of MAZ [38]. Here, we demonstrated that an increase in expression and endogenous transactivation of PPARγ1 in MCF-7 breast cancer cells enhances cell proliferation by accelerating cell transition from G_1 _to the S phase. This data was confirmed using a dominant-negative PPARγ1 mutant as an alternative approach to inhibit endogenous activity of PPARγ1 in two different cell lines, MCF-7 and T47D. We also found that in the absence of exogenous stimulation high expression of PPARγ1 significantly inhibits apoptosis in MCF-7 cells.

## Results

### PPARγ1 is highly expressed in breast cancer cell lines

**Figure 1:**
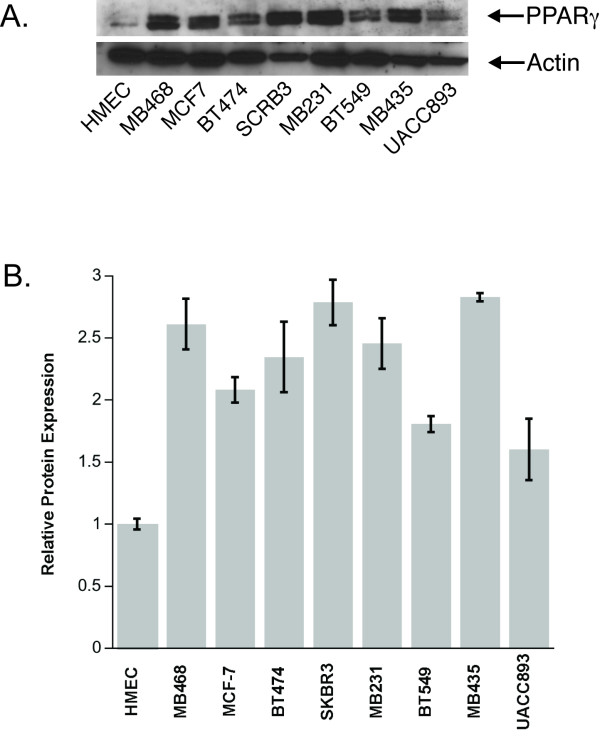
**PPARγ1 is over-expressed in various breast cancer cell lines as compared to HMEC**. **A**. The representative Western blot of PPARγ1 expression. Whole cell lysates (40 μg of total protein) from nine different cell lines were analyzed by Western blot analysis as described in the methods section. **B**. Densitometry was used to quantify PPARγ1 expression. The chart represents data from the three different immunoblots (n = 3). Intensity of each band was normalized to actin. PPARγ1 expression is shown as a fold change in band intensity relative to HMEC. Statistical analysis was performed and demonstrated a significant difference in PPARγ1 expression in all tested cancer cell lines as compared to HMEC (p < 0.05).

Our previous studies revealed that MAZ is a critical transcriptional regulator of PPARγ1 in MCF-7 breast cancer cells [37]. We also showed that both PPARγ1 and MAZ are highly expressed in MCF-7 cells as compared to normal mammary epithelial cells (HMEC) and tumor-specific expression of PPARγ1 is MAZ dependent [37]. To evaluate expression of PPARγ1 within other breast cancer cell lines, whole cell lysates from a panel of eight different breast cancer cell lines originated from heterogeneous tumors (ranging from adenocarcinoma to metastatic ductal carcinoma) were examined by Western blot analysis. HMEC cells were used as a control. Figure 1A shows the representative immunoblot for PPARγ1. To demonstrate the reproducibility of these data, statistical analysis of three Western blots was performed (Fig. 1B). The results showed that PPARγ1 is expressed at significantly higher level in cancer cell lines as compared to HMEC.

### RNAi effect on PPARγ1 expression and activity in MCF-7 breast cancer cell line

**Figure 2:**
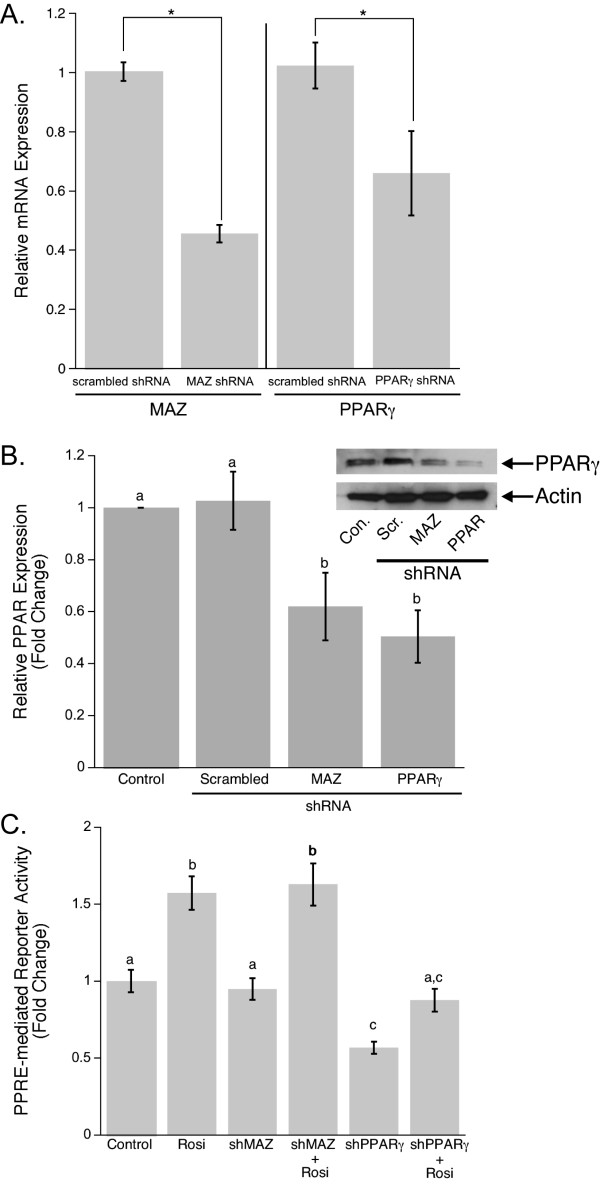
**Effect of PPARγ and MAZ shRNAs applications on down-regulation of PPARγ1 expression and activity in the MCF-7 breast cancer cell line**. **A**. To test the specificity of MAZ and PPARγ shRNAs for their target genes and estimate the efficiency of MAZ and PPARγ knock-down, Real-time PCR analysis of MCF-7 cells transfected with scrambled, MAZ, or PPARγ shRNA was performed. The fold change in gene expression was calculated using the ΔΔCt method. 18S was included as an internal control.**B**. Representative Western blot analysis of PPARγ1 expression in MCF-7 cells transiently transfected with scrambled, MAZ, or PPARγ shRNA. Densitometry was used to quantify PPARγ1 expression (n = 3). PPARγ1 expression is shown as a fold change in band intensity relative to control MCF-7 cells. Intensity of each band was normalized to actin. **C**. PPRE-mediated reporter activity was measured in MCF-7 cells transiently transfected with a 3XPPRE-mTK-pGL3 reporter plasmid and then co-transfected with MAZ or PPARγ shRNA expression plasmids. Cells were also subsequently treated with 10 μM Rosi for 20 hours. Data is expressed as mean fold change in luciferase to renilla ratios compared to control.

As discussed above, evaluation of PPARγ1 as a potential breast cancer therapy target revealed the complexity of PPARγ1 signaling in cancer. The mechanism of PPARγ1 activation during cancer development and the functional role of this event in tumorigenesis still remain unclear. To address these questions and elucidate the role of PPARγ1 activation in cancer, we utilized the advantages of shRNA techniques. A set of five shRNAs for each of PPARγ or MAZ gene was purchased from The RNAi Consortium (TRC). Each shRNA was evaluated using Western blot analysis (data is not shown). The most efficient shRNA for each gene was chosen for further investigation. MCF-7 cells were transiently transfected with PPARγ and MAZ shRNAs, as well as with a scrambled shRNA as control. MCF-7 cells treated only with the transfection reagent were also used as a control. To evaluate the specificity of shRNAs to their target genes and the extent of PPARγ1 down-regulation when either PPARγ or MAZ shRNA were applied to MCF-7 cells, Real-time PCR, Western blot analysis and Luciferase assay were performed. Real-time PCR data revealed that both PPARγ and MAZ shRNAs are highly specific to their targets and their application to the cells leads to a significant decrease in PPARγ1 or MAZ mRNA levels respectively (Fig. 2A). We also tested whether the observed changes in PPARγ1 or MAZ mRNA lead to changes in PPARγ1 protein expression. Figure 2B shows the representative immunoblot for PPARγ1 and demonstrates the level of PPARγ1 down-regulation by both PPARγ and MAZ shRNAs as compared to controls. The statistical evaluation of three different Western blots showed that the direct inhibition of PPARγ1 by PPARγ shRNA resulted in an average 50 percent decrease in PPARγ1 expression. A lower level of inhibition was observed when PPARγ1 knocked-down was achieved via down-regulation of MAZ expression. This was anticipated since we believe transcription factors other than MAZ are involved in regulation of PPARγ1 in cancer cells.

It is known that PPARγ activates gene transcription by interacting with a Peroxisome-Proliferator Response Element (PPRE) located within the promoter sequence of target genes [2]. To confirm that shRNAs-mediated down-regulation not only affects expression but also activity of PPARγ1, a PPRE functional response was measured. MCF-7 cells were transfected with a 3XPPRE-mTK-pGL3 reporter plasmid and then co-transfected with scrambled, PPARγ, or MAZ shRNA expression plasmids. Following transfection, cells were treated with 10 μM Rosiglitazone (Rosi), a well known PPARγ agonist [39]. Cells were lysed 24 hours after the second transfection and a Luciferase assay was performed. Data demonstrated that direct down-regulation of PPARγ1 expression by PPARγ shRNA led to a significant decrease in PPRE-mediated reporter activity in both Rosi treated and untreated MCF-7 cells (Fig. 2C). This confirms that PPARγ shRNA is specific and efficient for the inhibition of PPARγ1 expression and activity. Moreover, the fact that PPRE activity falls below the control level when PPARγ shRNA is applied to the cells is additional evidence for endogenous transactivation of PPARγ1 in breast cancer cells. Although we observed a decrease in reporter activation when MAZ shRNA was transfected to the cells, these changes were not statistically significant, suggesting the complexity of PPARγ transcriptional regulation and that the knock-down of MAZ seen in transient transfection assays is not sufficient to block PPRE-mediated reporter activity.

### Down-regulation of PPARγ1 expression by PPARγ or MAZ shRNA decreases proliferation of MCF-7 breast cancer cells

**Figure 3:**
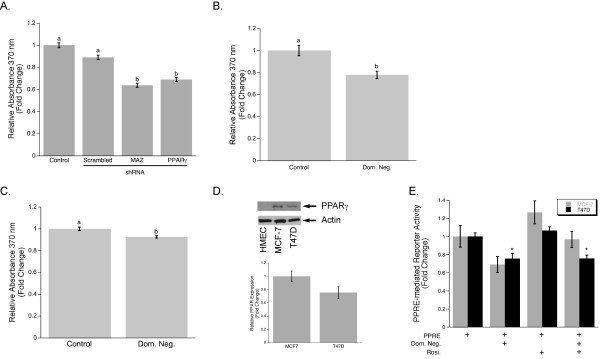
**Decreased PPARγ1 expression is associated with a less proliferative phenotype for MCF-7 cells**. **A**. Cell proliferation of MCF-7 cells transfected with or without scrambled, MAZ, or PPARγ1 shRNA was measured based on incorporation of the pyrimidine analog BrdU into DNA (BrdU proliferation ELISA). Data is shown as mean fold changes in cell proliferation compared to control cells. Error bars represent the standard error of the mean (s.e.m.) and the bars that do not share a letter designation were determined to be significantly different by Tukey's pairwise comparison (p < 0.05). **B**. MCF-7 cells were transiently transfected with a Δ462 expression plasmid or control plasmid. Cell proliferation was measured by BrdU proliferation ELISA. **C**. T47D cells were transiently transfected with a Δ462 expression plasmid or control plasmid. Cell proliferation was measured by BrdU proliferation ELISA. In Fig. B and Fig. C Student's t-test showed a significant difference (p < 0.01). **D**. The level of PPARγ1 expression in HMEC, MCF-7, and T47D cells was determined using Western blot analysis. Densitometry was used to quantify PPARγ1 expression in MCF-7 and T47D (n = 3). PPARγ1 expression in T47D is shown as a fold change in band intensity relative to MCF-7 cells. Intensity of each band was normalized to actin. **E**. PPRE-mediated reporter activity was measured by Luciferase assay in MCF-7 and T47D cells transfected with a dominant-negative mutant, Δ462, or control plasmid. Cells were also subsequently treated with 10 μM Rosi for 20 hours. Data is shown as mean fold change in cell proliferation compared to control cells. Error bars represent the standard error of the mean (s.e.m.). * Significantly different from appropriate control at p < 0.01.

Since one of the most important characteristics of tumor development is enhanced cell growth, we tested whether inhibition of PPARγ1 expression affects cellular proliferation in breast cancer cells. MCF-7 cells were transiently transfected with scrambled, PPARγ, or MAZ shRNA and the rate of BrdU incorporation during DNA synthesis was assessed by using the BrdU proliferation assay (Roche). The results revealed that down-regulation of PPARγ1 by both PPARγ and MAZ shRNAs significantly decreased cellular proliferation in MCF-7 breast cancer cells (Fig. 3A). To confirm these results, we used a different approach to inhibit endogenous activity of PPARγ1. MCF-7 cells were transiently transfected with a vector driving the expression of a dominant-negative mutant of PPARγ, Δ462, or an empty vector as control, and then a BrdU proliferation assay was performed. Inhibition of endogenous PPARγ1 activity using the Δ462 mutant caused a decrease in cellular proliferation in MCF-7 cancer cells (Fig. 3B). To test whether endogenous activation of PPARγ1 plays a similar role in other types of breast cancer cells, the same experiment was performed using the breast cancer cell line, T47D. The level of PPARγ1 expression in this cell line was evaluated using Western blot analysis (Fig. 3D). T47D cancer cells were transiently transfected with either a control vector or a Δ462 expression vector and then a BrdU proliferation assay was performed. Inhibition of PPARγ1 activity in these cells also led to a significant decrease in cellular proliferation (Fig. 3C). To confirm that the observed changes in cellular proliferation in both MCF-7 and T47D cell lines are indeed in response to the inhibition of PPARγ1 activity in a dominant-negative manner by Δ462, a Luciferase assay was performed. Cells were transfected with 3XPPRE-mTK-pGL3 reporter plasmid and then co-transfected with control or Δ462 expression plasmids. Following transfection, cells were treated with 10 μM Rosi. In Rosi treated and untreated cells, application of a Δ462 mutant resulted in a significantly lower level of PPRE-mediated reporter activity (Fig. 3E), thus, demonstrating that Δ462 efficiently inhibits endogenous activity of PPARγ1 in MCF-7 and T47D cancer cells.

### Down-regulation of PPARγ1 gene expression affects cell cycle distribution by decreasing the number of cells entering S-phase in MCF-7 cells

**Figure 4:**
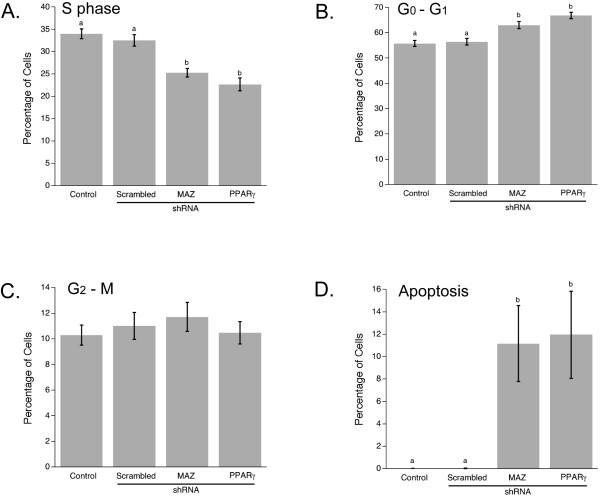
**Down-regulation of PPARγ1 or expression of MAZ prevents S-phase entry in MCF-7 breast cancer cells**. Changes in cell cycle distribution of MCF-7 cells transfected with or without scrambled, MAZ, or PPARγ1 shRNA were analyzed by FACS. Cells were stained with propidium iodide (PI). Mean and s.e.m. from five independent experiments are shown. Error bars that do not share a letter designation were determined to be significantly different by Fisher's LSD pairwise comparison (p < 0.05). **A**. Inhibition of PPARγ1 or MAZ expression leads to a decrease in a number of cells entering S-phase. **B**. A decrease in the number of proliferating cells in PPARγ1 or MAZ shRNA transfected cells is associated with an increase in the number of G_0_-G_1 _arrested cells. **C**. There was no significant difference in M-G_2 _phase cell number observed among all groups. **D**. Down-regulation of PPARγ1 as well as MAZ leads to an increase in the number of apoptotic cells.

To elucidate the mechanism by which down-regulation of PPARγ1 expression leads to inhibition of cellular proliferation in MCF-7 cells, fluorescence-activated cell sorting (FACS) was performed. Analysis of cell cycle distribution (Fig. 4) revealed that PPARγ1 down-regulation by PPARγ or MAZ shRNA primarily affects cell transition from G_1 _to S-phase in MCF-7 cells. Approximately 25 percent fewer cells entered the S-phase when PPARγ1 expression was suppressed directly or indirectly. This is consistent with data from the BrdU proliferation assay. Therefore, these results confirm our hypothesis and demonstrate that an increase in PPARγ1 expression and its endogenous transactivation play an important functional role in promoting cellular proliferation in breast cancer cells.

FACS analysis allowed us to assess changes in apoptosis as well. Interestingly, the level of apoptosis (Fig. 4D) and the percentage of debris and aggregates (data not shown) in cells transfected with either PPARγ or MAZ shRNA was significantly higher than in control cells or scrambled shRNA transfected cells. This observation suggests that in addition to its involvement in regulation of proliferation, PPARγ1 may also be involved in regulation of apoptosis in MCF-7 cells.

### Down-regulation of PPARγ1 gene expression increases apoptosis in MCF-7 breast cancer cells

**Figure 5:**
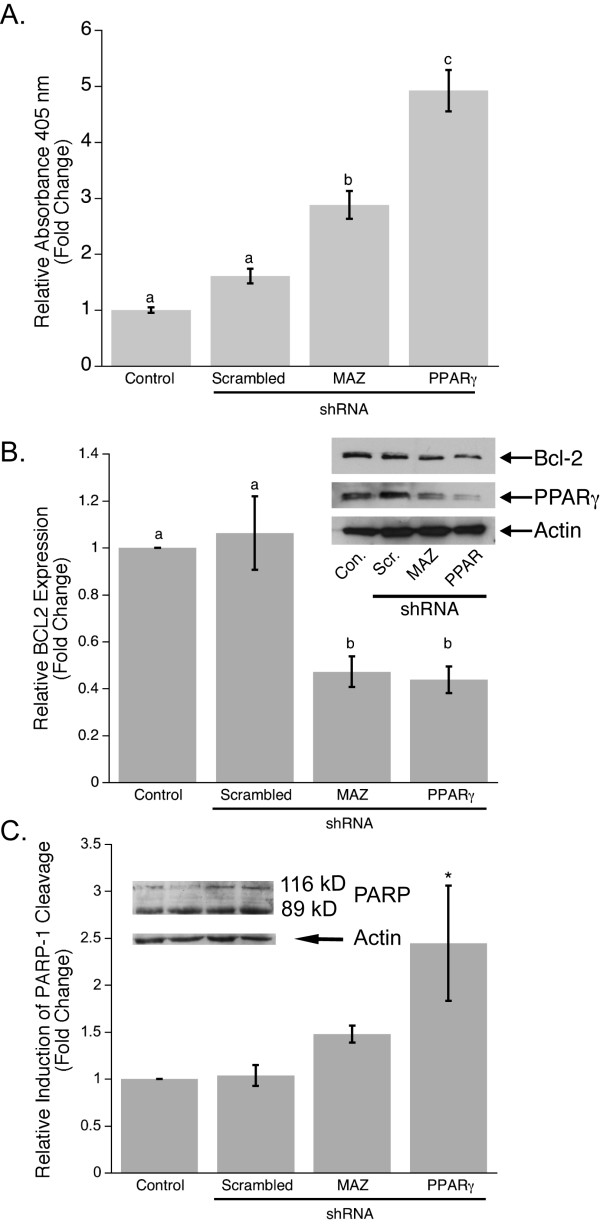
**Down-regulation of PPARγ1 increases apoptosis in MCF-7 breast cancer cells**. **A**. Apoptosis was measured by specific determination of mono- and oligonucleosomes in the cytoplasmic fraction of cell lysates from control and shRNA transfected MCF-7 cells. Data is shown as mean fold changes in cell apoptosis compared to control. Error bars represent s.e.m. and the bars that do not share a letter designation were determined to be significantly different by Tukey's pairwise comparison (p < 0.05). **B**. Representative Western blot analysis of PPARγ1 and Bcl2 expression in control and shRNA transfected MCF-7 cells showed that a decrease in PPARγ1 expression leads to a decrease in Bcl2 expression. Densitometry was used to quantify Bcl2 expression (n = 4). Bcl2 expression is shown as a fold change in band intensity relative to control MCF-7 cells. Intensity of each band was normalized to actin. **C**. Western blot analysis of PARP-1 demonstrated an increase in induction of PARP-1 cleavage in MCF-7 cells transfected with PPARγ1 shRNA. Densitometry was used to quantify an 89 kDa fragment of PARP-1 cleavage (n = 3). It is shown as a fold change in the 89 kDa band intensity relative to control MCF-7 cells. Intensity of each band was normalized to actin. * Significantly different from control at p < 0.05.

To evaluate data from FACS analysis (Fig. 4D) and determine whether changes in PPARγ1 expression can affect apoptosis, a Cell Death Detection ELISA assay was performed which distinguishes between necrotic and apoptotic cell death. MCF-7 cells were transfected with scrambled, PPARγ, or MAZ shRNA using the same transfection protocol and time points as for the proliferation assay, FACS, and protein analysis. The results showed no significant difference in necrotic cell death between PPARγ knock-down and control cells (data not shown). However, inhibition of PPARγ1 expression in MCF-7 cells using PPARγ or MAZ shRNA resulted in a significant increase in apoptosis as compared to control (Fig. 5A). Interestingly, changes caused by down-regulation of PPARγ1 directly by using PPARγ shRNA were more dramatic than through knock-down of MAZ. This suggests that MAZ probably contributes to regulation of apoptosis mostly through its mediation of PPARγ1 signaling in cancer cells. To confirm that down-regulation of PPARγ1 expression induces apoptosis, the expression of Bcl2, an anti-apoptotic protein, which has been shown to promote the survival of cancer cells [40] was evaluated. Statistical analysis of densitometry for four Western blots demonstrated that Bcl2 expression was significantly higher in control and MCF-7 cells transfected with scrambled shRNA versus cells transfected with PPARγ or MAZ shRNA (Fig. 5B). We also assessed changes in poly (ADP-ribose) polymerase-1, PARP-1, cleavage in cells transfected with PPARγ or MAZ shRNA as compared to controls. PARP-1 cleavage by caspases is a well-known marker for apoptosis [41]. Statistical evaluation of densitometry for three Western blots (Fig. 5C) showed a significant increase in an 89 kDa C-terminal fragment, the product of PARP-1 proteolysis, when PPARγ shRNA was applied to MCF-7 cells. This data suggests that an increase in PPARγ1 expression followed by transactivation during cancer development may be an important factor that contributes not only to acceleration of cellular proliferation but also to cell evasion from apoptosis.

## Discussion

In this report, we confirmed that PPARγ1 is highly expressed in cultured breast cancer cell lines as compared to HMEC [37,42]. High expression of PPARγ1 has also been reported in human breast cancer tissues [43]. However, questions about the mechanism and role of endogenous transactivation of PPARγ1 during development of breast cancer still remain unanswered. We have previously shown that the increase in expression of PPARγ1 from normal human mammary epithelia to breast cancer is due to the recruitment of a distal, tumor-specific promoter [34]. We identified MAZ as a transcription factor that directly binds to this promoter and drives expression of PPARγ1 in MCF-7 breast cancer cells [37]. Our results also indicated that MAZ is highly expressed in MCF-7 cells as compared to HMEC [36]. In this study, statistical analysis of three different Western Blots demonstrated an increase in PPARγ1 expression in a panel of different breast cancer lines and confirmed that it is a feature attributed not only to MCF-7 cells but also to other tested breast cancer cell lines. This observation suggests that the proposed model of endogenous PPARγ1 transactivation may apply not only to a particular cell line, but also to breast cancer in general. Currently this hypothesis is being tested in the lab using pathological sections from normal and breast cancer specimens.

In efforts to explore the role of PPARγ activation in cancer, most of the recent studies employed pharmacological approaches. The anti-cancer activity of PPARγ ligands, such as TZDs, demonstrated in multiple *in vitro *studies, has raised discussion about the possibility of using PPARγ receptors as a target for breast cancer therapy. However, the "off target" effects of PPARγ agonists [44,45], the dual role that some ligands play when they are applied to the cells at different concentrations [46], and the paradoxical anti-cancer effect of PPARγ antagonists [47] necessitated the use of other approaches to evaluate the consequences of PPARγ transactivation in cancer. For the first time, using an *in vitro *model, we have addressed questions about the role that endogenous transactivation of PPARγ1 plays in the pathogenesis of breast cancer. Using RNAi techniques to inhibit PPARγ1 expression we demonstrated that an increase in PPARγ1 signaling can significantly affect proliferation and apoptosis in breast cancer cells. It is widely accepted that the dysfunctional balance between cellular proliferation and apoptosis can contribute to the initiation and progression of cancer. Here, we demonstrated that down-regulation of PPARγ1, directly or indirectly via knock-down of its transcriptional regulator MAZ, leads to a decrease in cellular proliferation in MCF-7 breast cancer cells. Interestingly, changes in cellular proliferation caused by direct PPARγ1 inhibition by PPARγ shRNA were analogous to changes in PPARγ1 expression when inhibited via down-regulation of MAZ. This suggests that in addition to its role as a mediator of tumor-specific expression of PPARγ1, MAZ may also be involved in the regulation of other growth control genes in MCF-7 breast cancer cells. The ongoing project in our lab is to further investigate the role of MAZ in breast cancer development.

The observed pro-survival effect of PPARγ1 signaling in MCF-7 cancer cells was also confirmed by using a different approach to inhibit endogenous activity of PPARγ1. We took advantage of a PPARγ1 mutant, Δ462, which lacks helix12, critical for ligand binding and co-activators recruitment. Thus, Δ462 functions in a dominant-negative manner. Data from BrdU proliferation assays demonstrated that inhibition of PPARγ1 activity using Δ462 decreases cell proliferation not only in MCF-7 cells but in another widely studied breast cancer cell line, T47D. In our previous study, we have shown that the T47D cell line also has a functional peroxisomal response [42]. Here, using Western blot analysis, we demonstrated that T47D cancer cells as well as MCF-7cells have high level of PPARγ1 expression as compared to HMEC. However, the direct comparison of PPARγ1 expression in MCF-7 and T47D cells showed the lower level of PPARγ1 in a latter cell line. The differential expression of PPARγ1 in these cell lines can explain the more prominent changes in cellular proliferation in MCF-7 cells compare to T47D cells when Δ462 are applied to the cells. The specificity of Δ462 in the inhibition of endogenous PPARγ1 activity was confirmed using Luciferase assay. We measured PPRE-mediated reporter activity when either MCF-7 or T47D cells were transfected with Δ462 or control plasmids and then treated with10 μM Rosi. Data revealed that PPRE reporter activity is significantly lower in cells transfected with the Δ462 expression plasmid compared to control, thus, providing the evidence that this mutant acts in dominant-negative manner, decreasing activity of PPARγ1 in MCF-7 and T47D cancer cells. Moreover, a similar effect was observed with Rosi, confirming the specificity of Δ462 action in these cell lines. In summary, these results suggest that PPARγ1 transactivation enhances cell growth in breast cancer cells and that this phenomenon is not specific to MCF-7 cells.

To further investigate the mechanism by which PPARγ1 regulates cell growth, we performed fluorescence-activated cell sorting (FACS). Cell cycle distribution analysis confirmed results from the BrdU proliferation assay and demonstrated that an increase in PPARγ1 signaling accelerates the transition of cells from G_1_-phase to S-phase and, thus, increases cellular proliferation.

Blockage of apoptosis is a likely requirement for cancer maintenance [48]. In fact, FACS analysis revealed that the number of cells which undergo apoptosis is much higher in MCF-7 cells with decreased PPARγ1 expression. This observation was tested and confirmed by measuring DNA fragmentation in control and PPARγ or MAZ shRNA transfected cells. The data demonstrated that down-regulation of PPARγ1 expression in MCF-7 cells leads to a significant increase in apoptosis. The induction of apoptosis in cells with PPARγ1 or MAZ knockdown was also confirmed by analyzing the expression of Bcl2, a protein that is known to block cell death [40], and by evaluation of PARP-1 cleavage, a widely accepted marker for apoptosis. The results showed that inhibition of PPARγ1 leads to down-regulation of Bcl2 which may in turn favor re-activation of signaling pathways to induce apoptosis. The increase in PARP-1 cleavage in MCF-7 cells, which have a decreased level of PPARγ1 expression, verified the induction of apoptosis as well. Together these results suggest that accelerated PPARγ1 signaling can interfere with apoptotic pathways and promote cancer cell survival during breast tumor development. However, the molecular mechanisms that drive these events are not known and will be the subject of future investigation in the lab.

In summary, this study demonstrates that the increase in PPARγ1 expression observed in breast cancer results in an increase in PPARγ1 signaling that in turn promotes proliferation and inhibits apoptosis and thus, may significantly contribute to the progression of disease to a more malignant stage. Our findings are consistent with results from a study that evaluated the consequences of intrinsic PPARγ1 activation using transgenic mice. This study demonstrated that constitutive over-expression of PPARγ1 in mice, which were predisposed to breast cancer, leads to a greater number of tumors and higher mortality in both male and female animals, thus suggesting that increased PPARγ1 signaling serves as a tumor promoter in the mammary gland [31]. Since constitutive PPARγ1 signaling did not affect mammary gland differentiation or function when introduced in wild-type mice, the authors emphasized that consequences of PPARγ1 transactivation are different in normal and transformed cells. This observation is consistent with our previous data, which demonstrated different mechanisms of transcriptional regulation of PPARγ1 in breast cancer cells as compared to HMEC [34,37].

## Conclusion

This study provides insight into the functional significance of increased PPARγ1 expression and endogenous transactivation in breast cancer in an *in vitro *model. The results suggest that increased PPARγ signaling can act as a pro-survival factor by enhancing cancer cell proliferation and blocking the ability of the cell to undergo apoptosis. Furthermore, modulation of the PPARγ1 signaling pathway remains a promising tool for breast cancer therapy. The findings presented in this paper warrant further investigation regarding the use of PPARγ1 ligands, such as TZDs, in patients who are predisposed or already diagnosed with breast cancer. However, more broad and detailed studies are required to evaluate the impact of PPARγ1 signaling in breast cancer progression.

## Methods

### Cell culture

MCF-7 and T47D breast cancer cells were obtained from the American Type Culture Collection (Rockville, MD). Cells were cultured in modified DMEM (Gibco BRL, Gaithersburg, MD) supplemented with 10% fetal bovine serum (Hyclone). Normal human mammary epithelial cells (HMEC) (Cambrex) were cultured in MEGM^® ^with SingleQuot^® ^supplements. All cell lines were grown in media lacking phenol red at 37°C in a 5% CO_2 _atmosphere.

### Western blot analysis

The whole cell lysates were prepared using passive lysis buffer. Concentrations were determined using a Bradford Assay (BioRad). 30 μg of total cell lysate per sample was run on a 10% or 12% SDS polyacrylamide gel. The proteins were transferred to a nitrocellulose membrane, blocked in 5% TBST, and incubated at 4°C overnight with primary antibody. The membrane was then washed and incubated for 4 hours with secondary IgG-HRP antibody. After incubation the membrane was washed, incubated with Chemiluminescence substrate (Pierce) for 5 min, and expose to film. The following primary antibodies were used: PPARγ mouse monoclonal IgG antibody 1:200 dilution (Santa Cruz Biotechnology, sc-7273), Bcl2 mouse monoclonal antibody 1:1000 dilution (Santa Cruz Biotechnology, sc-509), PARP-1 rabbit polyclonal antibody 1:1000 dilution (Santa Cruz Biotechnology, sc-2578). Appropriate secondary goat anti-mouse (Santa Cruz Biotechnology, sc-2055), or bovine anti-goat (Santa Cruz Biotechnology, sc-2378), or goat anti-rabbit secondary antibody 1:1000 dilution (Santa Cruz Biotechnology, sc-2004) antibodies 1:1000 dilution were applied. Anti-actin raised in rabbit 1:2000 dilution (Sigma, A 5060) and goat anti-rabbit secondary antibody 1:1000 dilution (Santa Cruz Biotechnology, sc-2004) were used to visualize actin. Western Blot Stripping Buffer (Pierce, # 21059) was used to restore membranes.

### shRNAs constructs

The set of five shRNAs for PPARγ1 (TRCN 0000001670-74) and MAZ (TRCN 0000015343-47) genes as well as scrambled shRNA and non-hairpin TRC controls were purchased from The RNAi consortium (TRC) Human shRNA Library (Open Biosystems). The shRNA construct includes a hairpin of 21 base pairs, a sense and antisense stem, and a 6 base-pair loop. Each hairpin sequence is cloned into a lentoviral vector (pLKo1). Based on structural evaluation and Western blot analysis the most efficient shRNAs for PPARγ1 (TRCN 0000001672) and MAZ (TRCN 0000015345) were chosen for transient transfections.

### Dominant-negative PPARγ1 construct

The dominant-negative PPARγ1 mutant was a kind gift of Dr. Stephen O'Rahilly and Dr. V. Krishna K Chatterjee, Department of Medicine, Addenbrook's Hospital, Cambridge, U.K. Sequence analysis revealed a single base deletion introducing a premature stop codon (5'-_1380 _GACAGAC**TGA**_1390_-3') leading to translation of protein truncated just before the AF-2 domain.

### Transfection assays

Cells were transiently transfected with 3.6 μg of pGL3 plasmid containing 3XPPRE-mTK-Luc and Renilla (Allred, 2005) per 24-well plate and then co-transfected with scrambled, PPARγ or MAZ shRNAs, Δ462, or control plasmids using FuGENE transfection reagent (Roche). 4 hours after transfection cells were subsequently treated with 10 μM Rosi for 20 hours. Cells were lysed in 80 μl of passive lysis buffer and treated according to manufacture's instructions (Promega dual luciferase assay kit). Luminometry was performed on a Berthold Technologies Lumat 9507 (Wildbad, Germany). Results were calculated as raw Luciferase units divided by raw Renilla units (RLU's). Data is presented as mean fold changes in treated cells as compared to control cells.

### Real-time PCR

Total mRNA was isolated using an RNeasy Mini Kit (Qiagen, CA) according to manufactures instructions. Real-time PCR was performed on total RNA using the TaqMan One-Step RT-PCR Master Mix Reagents Kit (Applied Biosystems). The pre-optimized primers and probes for MAZ, PPARγ1, and 18S were purchased from Applied Biosystems.

### BrdU proliferation assay

MCF-7 cells were seeded at 0.1 × 10^4 ^cells/well in 96-well tissue culture plates. Cells were transiently transfected on the second and third day using 0.05 μg of plasmid and 0.3 μl of FuGENE 6 transfection reagent (Roche) per well. Control MCF-7 cells were treated with FuGENE6 only. 16 wells per each shRNA and control were used. The same experimental set-up was used when cells were transfected with a dominant-negative form of PPARγ1, Δ462. The media was changed before the second transfection. Cell Proliferation ELISA, BrdU (colometric) (Roche) was performed on the fifth day according to the manufacture instructions.

### Apoptosis assay

The same protocol as for the proliferation assay was used to plate and transfect MCF-7 cells. A Cell Death Detection ELISA (Roche) was performed on the fifth day. The assay is based on quantitative sandwich-enzyme-immunoassay-principle and uses mouse monoclonal antibodies directed against DNA and histones. Cells were lysed in a 96-well plate, centrifuged, and 20 μl of supernatant was transferred into streptavidin-coated wells. A mixture of antibodies was added and the plate was then incubated for 2 hours. The unbound components were removed by washing. ABTS substrate was added and the amount of mono- and oligonucleosomes were measured photometrically using the ELISA-plate reader according manufacture instructions (Roche).

### Cell cycle analysis

The DNA content of control and shRNAs transfected MCF-7 cells was analyzed using a detergent-trypsin method (Vindelov, 1983). MCF-7 cells were seeded at 1 × 10^6 ^cells in 100 mm culture plates. Cells were transiently transfected on the second and third day with 6 μg of plasmid using 18 μl of FuGENE6 transfection reagent (Roche). On the fifth day the propidium iodide labeling procedure and fluorescence-activated cell sorting (FACS) using Mod FitLT V.3.1 software was performed (University of Kentucky Flow Cytometry Facility).

### Statistics

Data was analyzed by a two-way analysis of variance (ANOVA) using the StatServer 6.1(Insightful, Seattle, WA) from the server maintained by the University of Kentucky's Department of Statistics. In every 2-way ANOVA, Tukey's pair-wise comparison test was used post-hoc. P-values of less than 0.05 were considered to be significant. One-way ANOVA with Fisher's LSD or Tukey's pair-wise comparison post-hoc test were also used where appropriate. When appropriate, Student's t-test was also used for data analysis on Microsoft Excel.

## Competing interests

The authors declare that they have no competing interests.

## Authors' contributions

All authors significantly contributed in the design of the study, data interpretation, and manuscript drafts. YYZ carried out all experiments. RCS performed all statistical analyses and figure design. NKW and XW assisted with Δ462 and PPRE-Luciferase experiments. MWK coordinated this study.
